# Direct or indirect composite for restoring permanent first molars affected by Molar Incisor Hypomineralisation (MIH): a randomized clinical controlled trial

**DOI:** 10.1038/s41405-023-00165-5

**Published:** 2023-08-12

**Authors:** Abdulrhman Hakmi, Mayssoon Dashash

**Affiliations:** Department of Pediatric Dentistry, College of Dentistry, Damascus University, Damascus, Syria

**Keywords:** Composite resin, Bonded restorations, Paediatric dentistry

## Abstract

**Aim:**

This study was undertaken to compare direct composite resin restorations (DCRR) and indirect composite resin restorations (ICRR) for treating permanent first molars affected by MIH in terms of clinical performance.

**Materials and methods:**

This was a controlled, randomized, clinical split-mouth study. The studied sample consisted of 40 asymptomatic first permanent hypomineralised mandibular molars in 20 children aged between 7–11 years, these cases were divided randomly into two groups: Group 1 (experimental): 20 first permanent mandibular molars were restored with ICRR, and Group 2 (control): 20 first permanent mandibular molars that were restored with DCRR. The cavity was prepared using a diamond bur on a high-speed handpiece, and the prepared cavity was wiped with cotton moistened with sodium hypochlorite. The composite was applied directly with a total-etch bonding system. In the ICRR group, an impression for the prepared cavity was taken using a silicon-based material, and the restoration was adhesive with self-adhesive resin cement. The child’s satisfaction with each of the two application techniques was assessed through the scale FACES. Restorations were evaluated during follow-up periods (3, 6, and 12 months) according to Modified USHPH criteria.

**Results:**

The clinical success rate was 90% in the ICRR group versus 85% in the DCRR group after 12 months of follow-up without statistically significant differences (*P* = 0.218). Children were significantly more satisfied (*P* = 0.0351) with ICRR than DCRR.

**Conclusions:**

Both DCRR and ICRR can be considered effective restorations with acceptable clinical performance in the restoration of hypomineralised first permanent molars with an advantage of ICRR in terms of child acceptance of the restoration application technique.

## Introduction

Molar incisor Hypomineralisation (MIH) is defined as a developmental defect in teeth that primarily affects enamel in the first permanent molars with varying degrees of severity ranging mainly from mild enamel opacity to severe enamel break down, especially in occlusal regions and can involve the incisors [1]. This condition is considered a result of various environmental factors that affect the enamel from the pre and post-natal stage and continues until early childhood [2].

Prevalence of MIH is considered one of the highest enamel defects, ranging from 3 to 40% [3], Some studies reach 39.3% [4], so it is relatively common and a condition that would cause treatment challenges for clinicians due to severe sensitivity, breakdown of the occlusal surface, difficulty in cleaning, and relatively high failure [3]. Children with MIH show serious clinical management problems as they may have behavioral management issues, dental fear, pain, sensitivity, anxiety due to the appearance of their teeth, and bad experience because of multiple failed dental treatments [5].

Etiology for MIH is currently unknown, many factors and conditions may work together and increase the risk of its occurrence as some systemic and genetic factors work synergistically [6], prenatal or childhood illness is likely to be associated with MIH [7], Illness and birth complications are risk factors that can be controlled to prevent MIH [8].

According to Garot et al., prenatal factors (Generic “maternal illnesses”), perinatal factors (hypoxia at birth, cesarean, and prematurity) and postnatal factors (measles, otitis media, urinary tract infection and bronchitis, gastric disorders, fever, kidney diseases, pneumonia, asthma, antibiotic use) were shown to be significantly associated with MIH [9].

The evidence for management of MIH affected molars overall is weak. In mild cases, preventive approach is very important including oral hygiene instruction, dietary advice to both children and their careers, placement of topical fluoride varnish, and resin-based fissure sealants. In severe cases, the treatment is more complex, including restoration with composite resin, preformed metal crowns, laboratory manufactured indirect restorations, pulp therapy, and scheduled extractions [10, 11].

In the case of using full crowns, the need to preserve the remaining tooth structure can not be achieved when treating these teeth in children with relatively short clinical crowns, wide pulp chambers, greatly extended pulp horns and unstable contact points [12].

In dental restorations with the need for both durability and a conservative preparation, ICRR may be a possible solution for the treatment of hypomineralised permanent first molars and is midway between DCRR and full crowns [13]. On the other hand, indirect restorations are considered a conservative solution compared to full crowns and are characterized by greater control over laboratory procedures, systematic preparation, and reshaping of contact points [14].

ICRRs are an esthetic alternative to cast metal restorations and preformed metal crowns with minimal microleakage [15]. there is no evidence of which is the best restoration.

With the lack of clinical trials that compare the use of ICRR in severe degrees of MIH and other materials such as direct resin composite restorations, it is important to investigate the clinical outcome of ICRR when compared to direct restorations. The first null hypothesis was that DCRR and ICRR had no effect on the clinical performance of restored permanent first molars affected by MIH, and the second null hypothesis was that DCRR and ICRR had no effect on the child’s satisfaction with each of the two application techniques.

## Materials and methods

### Ethical considerations

The study protocol was approved by the Scientific Research and Postgraduate Board of Damascus University, Ethics Committee, Damascus University, Syria (IRB No. UDDS-3116-07092020/SRC-621). The trial was also registered on clinicaltrials.gov (NCT05299489). A detailed information sheet written in plain, non-technical language was provided in advance and the parent/guardian was asked to sign an informed consent form.

### Study population and inclusion criteria

The sample size was determined using the PS Power and Sample Size Calculation Program, version 3.0.43. Effect size (*d*) for secondary caries was considered for the calculation of the sample size, based on a previous study the effect size (d) for secondary caries was 0.9 [15]. Calculating the sample size yielded the required sample size of 16 first permanent molars per group to detect significant differences (significance level 5%, power 90%,). To compensate for the 20% dropout rate, the number was increased to 4 first permanent molars per group, with a total sample size of 40 first permanent molars per group.

A total of 40 hypomineralised first permanent molars of 20 patients were evaluated for the study and invited to participate in the study according to the following inclusion criteria:

- The diagnosis of MIH was based on the criteria proposed by the European Academy of Pediatric Dentistry (EAPD) [11].
- Children who were included to be part of this study were healthy and cooperative children according to Frankel’s scale (Scale 3 and 4).
- The children were aged between 7 and 10 years.
- Children who had two severe hypomineralised first permanent mandibular molars according to EAPD classification (Demarcated enamel opacities with breakdown and caries, spontaneous and persistent hypersensitivity affecting function e.g., brushing, mastication) that could be restored.
- No past dental treatment for the hypomineralised first permanent mandibular molars.
- Caries lesions that include the occlusal surface and do not exceed more than two-thirds of the dentin thickness in the periapical radiographic examination,
- Areas of hypomineralisation that include one-third of the affected tooth surface but less than two-thirds.
- No past dental treatment for the hypomineralised first permanent mandibular molars.
- Vital pulp according to chloroethyl test with no fistula or abscess, no story of spontaneous or continuous pain, and absence of clinical and radiographic signs of pulp necrosis (internal or external root resorption, movement of the tooth, and swelling).

While Exclusion criteria were related to:

- The presence of systemic disease.
- Children who had two mild hypomineralised first permanent mandibular molars according to EAPD classification (Demarcated enamel opacities without enamel breakdown Induced sensitivity to external stimuli e.g., air/water but not brushing, mild esthetic concerns on discolouration of the incisors).
- Any potential confounding enamel defects i.e., amelogenesis imperfecta.
- Uncooperative children according to Frankel’s scale (Scale 1 and 2).
- A history of allergic reactions to local anesthetics or some components of restorative materials.

All patient were assessed by 3 Pedodontists to ensure EAPD Diagnostic criteria of MIH, and all teeth were treated by one Pedodontist.

**Fig. 1:**
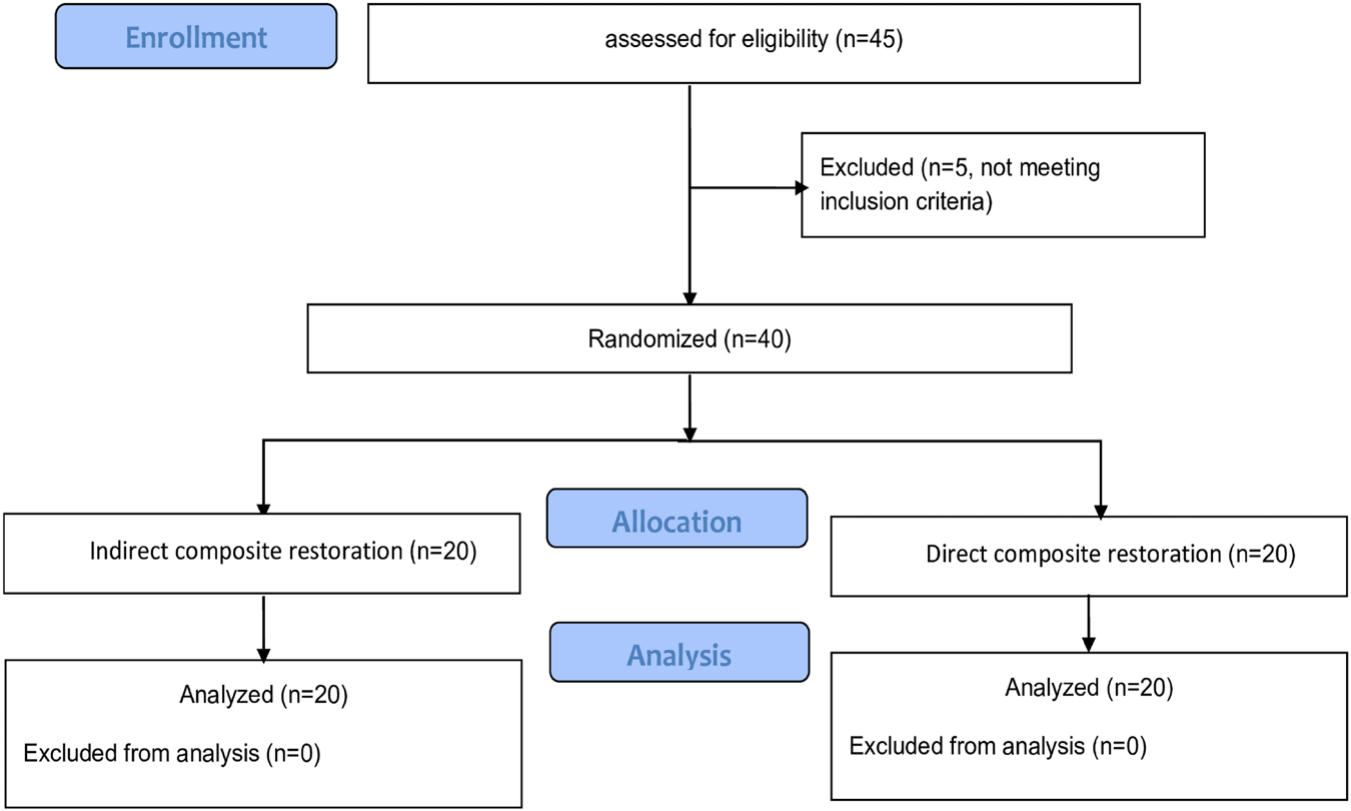
CONSORT flow diagram.

This trial has been designed according to CONSORT statement guidelines (Fig. 1) and the patients were randomly distributed automatically through the computer using the block randomization method, through randomization tables designed using the www.randomization.com, where the patients were randomly distributed into two random permuted blocks containing 20 patients with an allocation ratio of 1:1; Group (1, *n* = 20) represents the group in which the right side was treated with indirect resin composite as experimental restorations) and Group (2, *n* = 20) represents the group in which the right side was treated with direct resin composite as control restorations. A single-blinded study was also adopted so the examiners were masked about the applied materials.

### Intervention

**Fig. 2:**
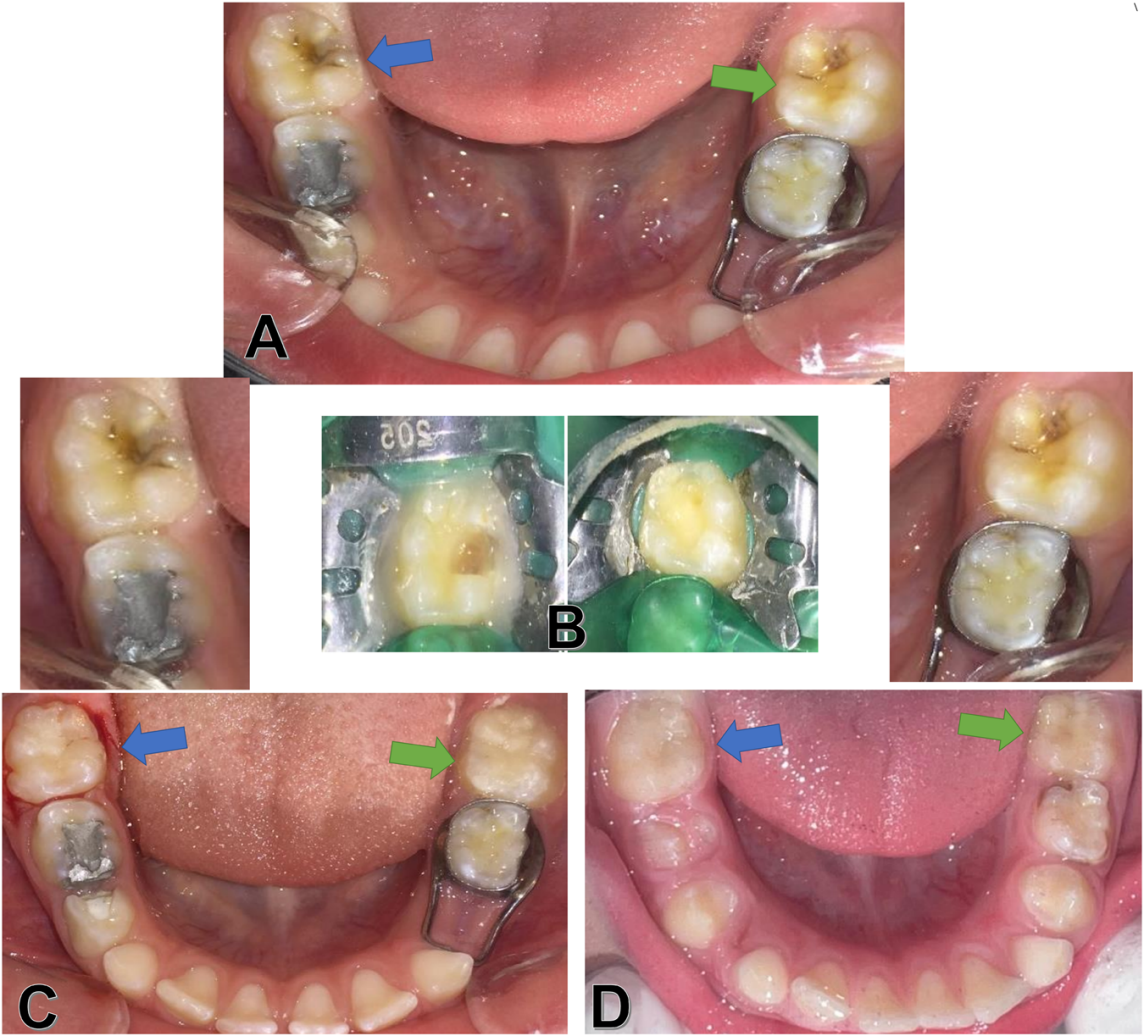
12-month follow-up of MIH first permanent molars restored with direct (green arrow) and indirect (blue arrow) composite: **A** Before treatment, **B** Isolation with a rubber dam and cavity preparation, **C** After treatment, **D** After 12 months.

All dental treatments were provided at the Department of Pediatric Dentistry, Faculty of Dentistry, Damascus University. The cavity was prepared after rubber dam isolation (Fig. 2B) and application of local anesthesia (2% lidocaine) using a diamond bur with a water-cooled high-speed handpiece. Dental caries was removed using an excavator on a slow-speed handpiece. The final margins of the preparation were placed on a sound enamel as Krämer et al.; showed poor bonding between hypomineralised enamel-resin composite and hypomineralised dentin-resin composite due to their high porosity [16], and if the remaining dentin thickness was less than 2 mm, a Glass Ionomer Cement (Fuji IX, GC Europe, Leuven, Belgium) base layer was placed.

All prepared walls were wiped with cotton moistened in sodium hypochlorite 5.25% before applying the acid etch and bond. Sodium hypochlorite enabled the removal of proteins from the infected MIH molars, which in turn can promote the inclusion of resin tags, which enhance the micro-mechanical bonding [17].

For ICRR, the preparation was made as the cavity walls were vertical to the longitudinal axis. The lingual and gingival margins of the proximal part were extended outside the contact areas by 0.5 mm, the functional cusps were reduced by 2 mm and then the non-functional cusps were reduced by 1.5 mm. The intervention was completed according to the application methods (direct or indirect):

*The DCRR group*: the phosphoric acid 37% (3 M Universal Etchant, 3 M Oral Care, USA) was applied to the enamel for 15 s, then to the enamel and dentin for 15 s, followed by thoroughly rinsing under running water for 10 s and dried with a gentle air. The single bond (3 M/ESPE) was placed on the enamel and was light cured for 20 s with a light-emitting diode light-curing unit (Elipar Freelight II, 3 MESPE). The resin composite (Filtek 350, 3M ESPE, St Paul, MN, USA) was applied in a layering technique, as the thickness of one layer does not exceed 2 mm, then each layer was light cured separately for 20 s. The finishing and polishing process was done using Fine (2135 F) and extra fine (2135FF) diamond burs (KG Sorensen), and then rubber points (8062, Viking; KG Sorensen) were applied until a smooth and polished surface was obtained

*The ICRR group*: teeth were cleaned after completing the preparation, and then the impressions were taken for both jaws using an additional silicone rubber impression material (Neosilk, Calmed Invest Kft, Busan, Korea). The impression was cast using yellow gypsum to obtain gypsum samples. Resin composite resin was applied to the gypsum samples in layering technique after they were isolated using silica, as the thickness of one layer does not exceed 2 mm, then each layer was light cured separately for 20 s. The finishing and polishing process was performed using Fine (2135F) and extra fine (2135FF) diamond burs (KG Sorensen), and then rubber points (8062, Viking; KG Sorensen) were applied until a smooth and polished surface was obtained.

The inner surfaces of the restoration were sandblasted using air abrasion with 50 μm aluminum oxide, then silane (Ceramic Bond Silane, Voco) was applied to the inner surface of the restoration with gentle air. A self-adhesive dual-cure resin cement (Breeze-Pentron Clinical) was applied using light-cured for 40 s from all sides to ensure that the light reached the full thickness of the resin cement.

All children were given oral hygiene instruction and topical fluoride varnish application during the follow-up sessions.

### Outcome assessment

**Fig. 3:**
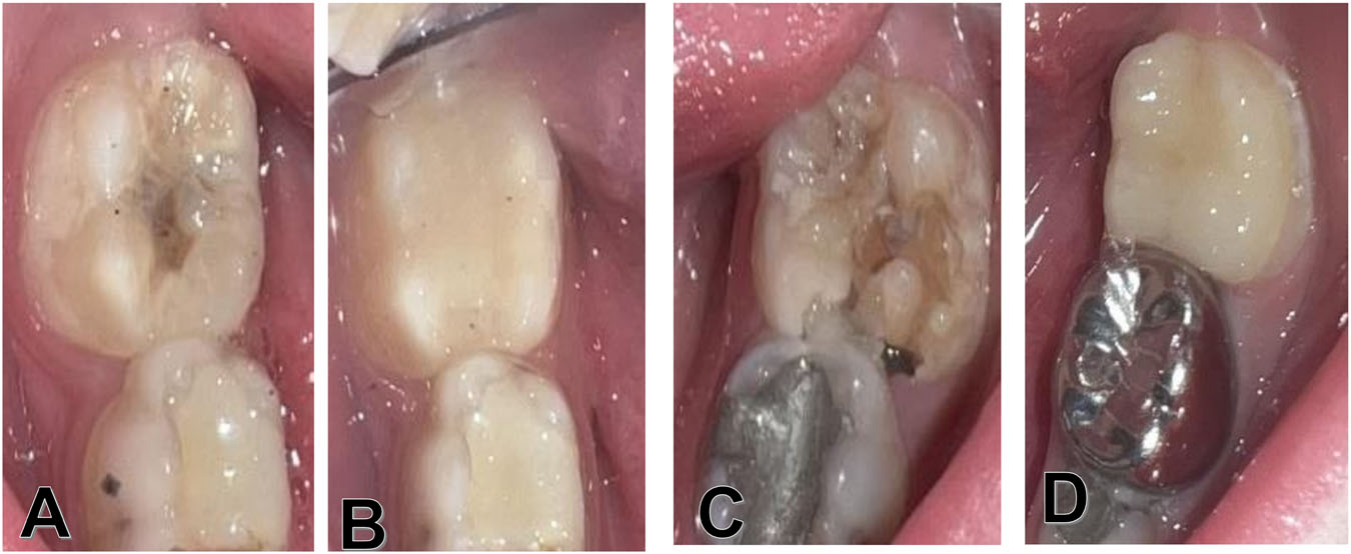
12-month follow-up of MIH first permanent molars restored with direct (**A** and **B**) and indirect (**C** and **D**) composite.

Restorations were evaluated during 3, 6, and 12 months (Figs. 2D and 3) by three Pedodontists to evaluate the success of the treatment according to the Modified United States Public Health Services criteria (USHPH) (Table 1) [18]. For analysis, restoration survival was considered successful when classified by alpha and bravo scores across all clinical conditions analyzed and failure was considered if one or more of these clinical conditions received a Charlie score.

**Table 1:** Modified USHPH criteria.

Criteria	Score	Clinical Situation
Anatomical form	Alfa	Continuous
	Bravo	Slight discontinuity, clinically acceptable
	Charlie	Discontinuous, failure
Marginal adaptation	Alfa	Closely adapted, no visible crevice
	Bravo	Visible crevice, the explorer will penetrate
	Charlie	Crevice in which dentin is exposed
Surface texture	Alfa	Enamel-like surface
	Bravo	Surface rougher than enamel, clinically acceptable
	Charlie	Surface unacceptably rough
Marginal discoloration	Alfa	No discoloration
	Bravo	Discoloration without penetration in the pulpal direction
	Charlie	Discoloration with penetration in the pulpal direction
Retention	Alfa	No loss of restorative material
	Charlie	Any loss of restorative material
Secondary caries	Alfa	No caries present
	Charlie	Caries present
Post-operative hypertensive	Alfa	No postoperative hypertensive
	Bravo	Moderate postoperative hypertensive
	Charlie	Severe postoperative hypertensive

The FACES scale comprises a row of five faces numbered from 1 to 5 and aims to assess state anxiety, the scores were recorded as follows: score 1 = Strongly agree (no anxious), score 2 = Agree (mild anxious), score 3 = Undecided (moderate anxious), score 4 = disagree (severe anxious), and score 5 = Strongly disagree (panic level anxiety) [19]. The children were asked to refer to the face that they felt most closely matched their feelings after ending treatment directly.

### Statistical analysis

Statistical analysis was performed by using the SPSS 21.0 software (IBM, Armonk, NY, USA). The data were analyzed with Mann Whitney U, paired t-test, and Chi-square test. The testing was performed at *α* = 0.05.

## Results

This study included 20 children aged 7–10 years (mean age = 8.3 ± 0.9) with MIH of which 11 were males (55%) and nine were females (45%) (Table 2). A total of 40 resin composite restorations were performed on the first mandibular molars where 92.5% (*N* = 37) of cases showed extreme sensitivity to cold, 82.5% (*N* = 33) post-eruptive breakdown, 85% (*N* = 34) atypical extensive caries (Table 3).

**Table 2:** Basic sample characters.

Gender		Ages			
Male	Female	Min	Max	Mean	SD
55%	45%	7	10	8.3	0.9

**Table 3:** shows the distribution of characteristics of FPMs per group.

	Permanent first molars N = 40				
		DCRR	ICRR	Total	*P-*value
No. of children *N* = 20	Extreme sensitivity^a^	18 (45%)	19 (47.5%)	37 (92.5%)	0.548
	PEB^a,b^	17 (42.5%)	16 (40%)	33 (82.5%)	0.673
	Atypical caries^a^	16 (40%)	18 (45%)	34 (85%)	0.375

^a^Based on Chi-Square test (*P* < 0.05).

^b^Post-eruptive breakdown.

Mann Whitney-U or Chi-Square tests were applied to pairwise comparison. After 12 months of follow-up, both DCRR and ICRR demonstrated acceptable anatomical form, marginal adaptation, surface texture, marginal discoloration, retention, secondary caries, and post-operative hypersensitivity with no statically significant differences (*P* = 0.287, 1.000, 0.106, 0.780, 0.584, 0.298) respectively. The survival rate of the two types of DCRR and ICRR was evaluated using Kaplan–Meier survival analysis, the survival rate was 90% in the ICRR group versus 85% in the DCRR group after 12 months of follow-up without statistically significant differences (*P* = 0.218) as seen in Table 4.

**Table 4:** Pairwise comparison between DCRR and ICRR during follow-up periods.

Criteria	Time interval						
	3 months		6 months		12 months		
	DCRR	ICRR	DCRR	ICRR	DCRR		ICRR
Anatomical form^a^							
Alpha	20 (100%)	20 (100%)	17 (85%)	19 (95%)	17 (85%)		19 (95%)
Bravo	0 (0%)	0 (0%)	3 (15%)	1 (5%)	2 (10%)		1 (5%)
Charlie	0 (0%)	0 (0%)	0 (0%)	0 (0%)	1(5%)		0 (0%)
* P*-value	_		0.298		0.287		
Marginal adaptation^a^							
Alpha	20 (100%)	20 (100%)	17 (85%)	18 (90%)	16 (80%)		16 (80%)
Bravo	0 (0%)	0 (0%)	3 (15%)	2 (10%)	3 (15%)		3 (15%)
Charlie	0 (0%)	0 (0%)	0 (0%)	0 (0%)	1 (5%)		1(5%)
* P*-value	_		0.218		1.000		
Surface texture^a^							
Alpha	19 (95%)	19 (95%)	15 (75%)	18 (90%)	12 (60%)		17 (85%)
Bravo	1 (5%)	1 (5%)	5 (25%)	2 (10%)	7 (35%)		2 (10%)
Charlie	0 (0%)	0 (0%)	0 (0%)	0 (0%)	1 (5%)		1 (5%)
* P*-value	_		0.218		0.106		
Marginal discoloration^a^							
Alpha	20 (100%)	19 (95%)	17 (85%)	19 (95%)	15 (75%)		16 (80%)
Bravo	0 (0%)	1 (5%)	3 (15%)	1 (5%)	5 (25%)		3 (15%)
Charlie	0 (0%)	0 (0%)	0 (0%)	0 (0%)	0 (0%)		1 (5%)
* P*-value	0.317		0.298		0.780		
Retention^b^							
Alpha	20 (100%)	20 (100%)	20 (100%)	20 (100%)	20 (100%)		20 (100%)
Charlie	0 (0%)	0 (0%)	0 (0%)	0 (0%)	0 (0%)		0 (0%)
* P*-value	_		_		_		
Secondary caries^b^							
Alpha	20 (100%)	20 (100%)	20 (100%)	20 (100%)	18 (90%)		19 (95%)
Charlie	0 (0%)	0 (0%)	0 (0%)	0 (0%)	2 (10%)		1 (5%)
* P*-value	_		_		0.548		
Post-operative hypertensive^a^							
Alpha	14 (70%)	16 (80%)	16 (80%)	18 (90%)	17 (85%)		19 (95%)
Bravo	6 (30%	4 (20%)	4 (20%)	2 (10%)	3 (15%)		1 (5%)
Charlie	0 (0%)	0 (0%)	0 (0%)	0 (0%)	0 (0%)		0 (0%)
* P*-value	0.496		0.382		0.298		
Survival rate evaluation^c^							
Success	20 (100%)	20 (100%)	20 (100%)	20 (100%)	17 (85%)	18 (90%)	
Failure	0 (0%)	0 (0%)	0 (0%)	0 (0%)	3 (15%)	2 (10%)	
* P*-value	_		_		0.636		

^a^Based on the Mann-Whitney U test (*p* < 0.05).

^b^Based on Chi-Square test (*p* < 0.05).

^c^Based on Kaplan–Meier test (*P* < 0.05).

Paired *t*-tests were applied to pairwise child satisfaction. Table 5 shows that children were more significantly satisfied (*P* = 0.0351) with ICRR (50% agree and 45% strongly agree), than DCRR (60% agree and 20% strongly agree).

**Table 5:** Pairwise child satisfaction between direct and indirect restorations.

Groups	Child satisfaction							
	Mean	SD**	Strongly agree score 1	Agree score 2	Undecided score 3	disagree score 4	Strongly disagree score 5	*P* value*
DCRR	2.05	1.60	4 (20%)	12 (60%)	3 (15%)	1 (5%)	0 (%)	0.0351
ICRR	0.76	0.60	9 (45%)	10 (50%)	1 (5%)	0 (0%)	0 (0%)	

^*^Based on Paired *t*-test test (*p*  <  0.05).

^**^SD Standard deviation.

## Discussion

The management of teeth with MIH lesions is a clinical problem due to its high prevalence, multiple clinical manifestations, young age of patients, severe dental sensitivity, and post-eruptive breakdown [20]. Although preformed metal and cast metal crowns are appropriate restorative options for severe MIH cases, the extensive preparation of complete crowns may not be a justification for the rehabilitation of all cases [21].

Scheduled extractions are also indicated for teeth with significant breakdown, or for those that are pulpally involved or associated with a dental abscess or facial cellulitis. In severe cases, consideration should also be given to the long-term prognosis of the tooth, the likelihood of repeated dental interventions and the psychological impact on the child [22]. Extraction may be the best option in these cases but complete spontaneous space closure is not guaranteed, even if performed at the ideal time of 8–10 years of age [23].

Clinical studies on the evaluation of the clinical performance of direct resin composite restorations are few and limited, hence this study was conducted to evaluate the clinical performance of indirect resin composite in the restoration of MIH first permanent molars during a 12-month follow-up period.

Resin composite was selected to restore MIH first permanent molars because it requires conservative preparation and is the first choice in restoring molars affected by MIH, especially if the lesion includes one or two surfaces [24]. ICRRs were adopted instead of ceramic restorations, as they allow their application with smaller thicknesses and the possibility of fixing it inside the mouth [13].

In the ICRR, the restorations were made by the dentist on the gypsum samples, and the adhesion was performed using self-adhesive resin cement, but selective enamel etching was undertaken for 30 s, where both Goracci et al., and de Goes et al., showed an increase in the bond strength after selective enamel etching [25, 26].

A self-adhesive resin cement was used to shorten the etching, washing, and drying stages as it has good mechanical properties, including its low solubility in oral fluids. It also has good flowability due to its low viscosity, and thus it achieves the lowest possible cement thickness between the restoration and the tooth surface [27]. This cement hardening begins with the transformation of monomers into polymers as soon as it is exposed to light, and the light-curing time is 40 s, and this process continues by the chemical method, and this ensures complete hardening of the cement [28].

The resin composite was applied in both application methods (direct and indirect) with a layering technique so that the thickness of the layer does not exceed 2 mm to harden the entire layer of the resin composite [29]. The inner surface of ICRR was sandblasted with 50 μm aluminum oxide at a distance of 10 ml and a pressure of 2.5 bar for 10 s, which leads to an increase in the roughness of the inner surface of the restoration and thus increases the bonding surface and bonding strength [30].

Silane was applied after sandblasting the inner surface of ICRR to increase the bond strength as an additional covalent interaction occurs between the methacrylate group in the resin cement and the silane particles during the hardening process [30]. An additional silicone was used as it is the most accurate impression material, is hydrophilic, and reduces problems resulting from lack of good isolation [31], which makes it particularly suitable in children for ICRR.

The results of this study supported the first null hypothesis, as there were no significant differences in terms of the clinical performance of DCRR and ICRR in the restoration of MIH first permanent molars. It is possible to apply the indirect resin composite in the restoration according to the results recorded in this study, which may help to overcome the problems associated with the application of direct restorations in children such as polymerization shrinkage and post-operative sensitivity [32].

Despite the need for an additional session in ICRR, the shortness in the duration of the treatment session compared to the DCRR in one was more desirable for the child, as the second null hypothesis was rejected. The results of the assessment of the child’s satisfaction with the ICRR technique were (50% agree and 45% strongly agree) higher than DCRR (60% agree and 20% strongly agree) and this plays a role in gaining the child’s cooperation for dental procedures [33]. Statistically significant differences were found in favor of ICRR, where reducing the treatment time session played an important role in evaluating the child’s acceptance of the treatment technique, and this in turn was reflected clinically in the child’s satisfaction with the treatment provided, which may favor these treatments in children.

No loss of restorative material was observed during follow-up periods in both techniques, this indicates that the resin restorations have good retention and stability towards masticatory forces, and this is consistent with the Dhareula et al. study which evaluated metal and indirect resin onlays in the rehabilitation of first permanent molars affected with MIH in children aged 8–13 years old, the study was performed on 30 children. 42 teeth were equally divided into two groups (*n* = 21)., and Lygidakis et al. study evaluated direct resin composite in the restoration of molars affected with MIH in children aged 8–10 years old [15, 34].

The presence of secondary caries was examined using the probe, and the results of this study showed there were no secondary caries in 90% of DCRR and 95% of ICRR after 12 months of follow-up without significant differences between these two types of restorations. This could be attributed to the removal of all affected enamel and the placing of the restoration margins on sound enamel. This is consistent with the study of de Souza et al. [20] which evaluated the clinical performance of resin composite restorations with two different adhesive systems for molars affected with MIH in children aged 6–8 years old according to the modified US Public Health Service (USPHS) criteria. The results of the present study also differed from the study of Sönmez and Saat in terms of secondary caries which evaluated deproteinization and different cavity designs on resin restoration performance in 95 MIH-affected molars in children aged 8–12 years. The difference may be attributed to the fact that we have used a self-adhesive bond, while Sönmez and Saat used the traditional bond system for DCRR [17]. Secondary caries for ICRR were consistent also with Dhareula et al. [15].

Comparing the anatomical form changes in the DCRR and ICRR, the results showed no change in the anatomical form of 85% and 95% in both DCRR and ICRR, respectively. This is consistent with the study of Dhareula et al. and Sönmez and Saat studies [15, 17]. Our findings were also different from those of de Souza et al., who were unable to evaluate 6 cases at the end of 12 months out of 21 cases, while in our study there were no withdrawal cases during the follow-ups [20].

As for the surface texture, the DCRR showed an enamel-like surface in 60% of cases compared to 85% for the ICRR without statistically significant differences. This can be explained by the fact that the ICRR contains a larger percentage of filler particles, which makes it less susceptible to surface changes [35, 36].

In this study, it was observed that there was no marginal discoloration and closely marginal adaptation in 75 and 80% of cases respectively, while in the ICRR group, the closely marginal adaptation and no marginal discoloration reached 80% with no statistically significant difference between the two groups. This could be attributed to the application of both resin composite restorations with a layering technique to overcome the polymerization shrinkage by reducing the C-factor [37].

Through the results of this study, it was observed that there was no post-operative sensitivity in the DCRR group 70%, 80%, and 85% during 3, 6, and 12 months respectively, compared to 80%, 90%, and 95% for the ICRR group. These results may be attributed to the strict adherence to rubber dam isolation and adhesive ICRR by dual-cure resin cement, which achieves better sealing of the dentin canals and bypasses the two stages of etching and drying of the dentin. The results of this study are consistent with Lygidakis et al., study regarding post-operative sensitivity [34], while the results differed with the Sönmez and Saat study and this can be attributed to the use of self-adhesive bond while the conventional bond system was used in this study [17].

This is the first study that evaluated ICRR in the first permanent molars affected with MIH. It has included good sample size and good follow-up periods, but we recommend in the future to conduct further studies with longer follow-up periods to confirm these results. Treatment decisions were made on a subjected criteria based on defect size, cooperation, patient needs, and this is the major limitation of the study. This is because there is not a standardized protocol.

## Conclusion

The results indicated that using DCRR and ICRR in the restoration of MIH first permanent molars is effective with the advantage of ICRR which resulted in child satisfaction due to shorter treatment sessions. Future studies are still required to confirm our findings with a larger sample size and further follow-up.

## Data Availability

The data that support the findings of this study are available from the corresponding author.
